# Summer Complaints of Children—Symptoms and Treatment

**Published:** 1879-08

**Authors:** 


*Symptoms.*—The attack usually begins with diarrhœa, but sometimes vomiting and purging commence simultaneously. The stomach rejects everything swallowed. The intervals are marked with great languor and distress. There is usually great thirst. The appetite is variable. After a time the discharges become thin, copious and offensive, but they do not have the healthy, fecal smell.

An ordinary diarrhœa is frequently caused by cold, improper food, etc., and is nature's method of cure. Good nursing will assist nature in her efforts, and there need be no anxiety for the little patient; but when the symptoms become alarming the treatment should not be delayed.

*Treatment.*—Have your druggist mix thoroughly four grains of calomel with about as much powdered sugar as would fill a thimble. After it is mixed divide into twelve powders. Give the child one of these powders—by putting it in the mouth dry—every one, two, or three hours, according to the symptoms. Any mother or nurse can judge near enough how often to give the powders. Two hours after the child has taken the first powder, the character of the discharges will begin to change. They will be streaked with fecal matter which will have a more healthy appearance and smell. Before the twelve powders are all given the discharges usually become natural, vomiting ceases, and the child rapidly convalesces. In addition to the medical treatment the child should wear a flannel undershirt made to cover the shoulders and arms, and a strip of flannel wrapped around the body so as to protect the bowels. A plaster of one-third mustard and two-thirds flour may be put over the stomach, a few minutes at a time, taking care that it does not blister. The feet should be kept warm with flannel cloths, heated before the fire, or dipped in hot water, and wrung nearly dry; or let the mother or nurse warm the hands and lay them gently over the child's stomach and bowels. The little patient should be kept in a cool, airy room, remote from the kitchen.

The *food* should be the mother's milk, or that of a healthy wet nurse, and the child should not be weaned during the existence of the complaint. The best substitute for this diet is fresh cow's milk, a very little diluted with water, for a child under six months. After six months pure rich milk should be given. Thousands of children have been starved to death with milk over diluted. In advanced stages of the disease, when the system is much enfeebled, it will be proper to allow a portion of animal food, such as chicken or mutton broth; or take a piece of lean, tender beef, chop it very fine, season with a little salt, and give it raw, a very little at a time. It is palatable, nutritious and digestible, and agrees with the stomach better than any other food. Van Deren recommends A TODDY, made by putting two to six teaspoonfuls of brandy or whisky into a quarter of a tumbler of cold water, adding a little sugar. Give one or two teaspoonfuls about once an hour. Whatever is given to the child must be given a little at a time, so as not to irritate the stomach and cause vomiting. Pounded ice, confined in linen or gauze, to prevent its being swallowed undissolved, will be found useful and very grateful to the little sufferer. Ice water may be given, a teaspoonful or two at a time, three or four times an hour. A little weak tea, with ice in it, is perhaps better. After recovery there is liability to relapses. The best way to avoid these is, if you reside in the city, take the child into the country and keep him there till cool weather. If in the country already, change the location, if you go only a mile away. I am acquainted with two physicians who always employ this plan of treatment, and they have had no fatal case out of the hundreds they have treated. This has also been my experience. Instead of calomel I use "VAN DEREN'S REMEDY." It is an excellent preparation, made in the form of little sugar-pellets, which any child will take readily, and does not require the caution in its use that calomel does. It is convenient and may be kept ready for an emergency. It costs only 50 cents a box, and a box will cure half a dozen children.

A single dose of 25 to 50 of these Van Deren sugar pellets, given as a cathartic to a child who has any malarial trouble will usually effect a cure. Indeed, I have never known of its failing to do so. I know of no remedy so generally applicable to the many ailments of children.

